# Regulation of Hydrogen Sulfide Metabolism by Nitric Oxide Inhibitors and the Quality of Peaches during Cold Storage

**DOI:** 10.3390/antiox8090401

**Published:** 2019-09-16

**Authors:** Biao Geng, Dandan Huang, Shuhua Zhu

**Affiliations:** College of Chemistry and Material Science, Shandong Agricultural University, Taian 271018, China; GB20160352@163.com (B.G.); ddhuang@sdau.edu.cn (D.H.)

**Keywords:** nitric oxide, hydrogen sulfide, peach, storage, metabolism, quality

## Abstract

Both nitric oxide (NO) and hydrogen sulfide (H_2_S) have been shown to have positive effects on the maintenance of fruit quality during storage; however, the mechanisms by which NO regulates the endogenous H_2_S metabolism remain unknown. In this experiment, peaches were immersed in solutions of NO, potassium 2-(4-carboxyphenyl)-4,4,5,5-tetramethylimidazoline-1-oxyl-3-oxide (c-PTIO, as an NO scavenger), N-nitro-l-arginine methyl ester (l-NAME, as an inhibitor of nitric oxide synthase (NOS)-like activity), and sodium tungstate (as an inhibitor of nitrate reductase), and the resulting changes in the H_2_S metabolism of peaches were studied. The results showed that exogenous NO reduced the contents of endogenous H_2_S, Cys, and sulfite; decreased the activities of l-/d-cysteine desulfhydrase (l-/d-CD), *O*-acetylserine (thiol)lyase (OAS-TL), and sulfite reductase (SiR); and increased the activity of β-cyanoalanine synthase (β-CAS). Both c-PTIO and sodium tungstate had similar roles in increasing the H_2_S content by sustaining the activities of l-/d-CDs, OAS-TL, and SiR. l-NAME increased the H_2_S content, mainly by maintaining the d-CD activity. The results suggest that NO, c-PTIO, l-NAME, and sodium tungstate differently regulate the H_2_S metabolism of peaches during storage.

## 1. Introduction

Hydrogen sulfide (H_2_S), as a bioactive signaling molecule, exhibits multiple functions in the various plant developmental stages and in their responses to different stresses, including heavy metal exposure, temperature, drought, and salt stress [1,2,3]. It is also considered to function as a signaling molecule during plant cross-adaptation [4]. Exogenous H_2_S, as well as donors such as sodium hydrosulfide (NaHS), can decrease Na^+^ concentration to alleviate growth inhibition caused by NaCl stress in wheat seedlings [5]. NaHS induces the antioxidant system and osmolyte biosynthesis to improve the seed germination and seedling growth of maize under elevated temperatures [6]. Environmental stresses cause the accumulation of endogenous H_2_S, which regulates the peroxisomal H_2_O_2_ metabolism, as a novel component in peroxisomes, by inhibiting catalase activity [7]. It has been reported that the physiological concentration of H_2_S ranges from 0.0089 to 1.978 μmol g^−1^ on the basis of fresh weight, independent of the species, organ, or developmental stage of *Arabidopsis* [8]. In plants, endogenous H_2_S is produced via the reduction of sulfite to sulfide, which is catalyzed by sulfite reductase and cysteine-dependent reactions [9]. l- and d-cysteine desulfhydrase (l-CD/d-CD), sulfite reductase (SiR), β-cyanoalanine synthase (β-CAS), and *O*-acetylserine (thiol)lyase (OAS-TL) are involved in the H_2_S metabolism of plants [10]. A minor concentration of H_2_S exerts signaling functions in physiological processes by interacting with reactive oxygen species (ROS) and nitric oxide (NO) [11,12]. NO induces the synthesis of H_2_S, maintains H_2_S levels and Cys homeostasis, and, in association with H_2_S, alleviates osmotic stress in wheat seedlings [13], barley seedling roots [14], maize [15], and pepper [16]. It is suggested that H_2_S acts either upstream or downstream of the NO signaling cascade depending on the process, e.g., stomatal closure or in response to abiotic stress [10]. 

It has also been confirmed that H_2_S regulates the growth and defense responses of plants, and even the postharvest physiology of horticultural crops [17], and inhibits the ripening and senescence of postharvest fruits [9,18]. Exogenous H_2_S inhibits the development of chilling injury in banana fruit by maintaining a high energy status [19], extending the postharvest life and enhancing antioxidant activity during storage [20]. It also alleviates postharvest ripening and the senescence of fruits by inhibiting ethylene production and antagonizing the effect of ethylene [21]. H_2_S is produced in plants, and the homeostasis of endogenous H_2_S is responsible for the alleviation of the senescence of postharvest products [22]. As an important bioactive molecule, NO enhances the storability and antioxidant systems, maintains the postharvest quality of fruits, and regulates fruit ripening [23,24,25]. The crosstalk between NO and H_2_S exhibits a synergistic inhibition of ethylene-induced fruit ripening [26], suggesting that the interplay between NO and H_2_S also plays important roles in fruit ripening [10].

Peach fruit is a large drupe with a skin-like exocarp, fleshy mesocarp, and thick endocarp, and is popular with customers due to its appealing appearance, rich juice, delicate pulp, and excellent taste. However, it perishes quickly at ambient temperature after harvest. Storage at low temperatures is a common method for prolonging the postharvest life of peach fruit. However, as a climacteric fruit, peach fruit easily suffers from chilling injury (CI) during cold storage, with internal browning, a loss of taste and aroma, and mealiness [27], which are unacceptable to consumers and lead to serious economic losses [28,29]. Both exogenous NO and H_2_S exhibit positive effects in inhibiting chilling injury, maintaining storage quality, and prolonging the shelf life of postharvest fruits [20,24,30,31]. However, there are few reports on the interplay between NO and H_2_S in postharvest fruit during storage. In this paper, the regulation of H_2_S metabolism by NO in peaches during cold storage was studied. 

## 2. Materials and Methods 

### 2.1. Plant Materials and Treatments 

Peach fruits (*Prunus persica* cv. (L.) Batsch, cv. “Xintaihong”) with a soluble solid content of about 12% and a firmness of about 110 N (Newtons) were harvested from Xintai, Shandong, China and precooled at 0 °C for 24 h. Peaches with uniform size and color and free from mechanical injury and diseases were immersed in one of the following solutions for 10 min: (1) Distilled water (control). (2) A 15 μmol L^−1^ NO solution. The NO aqueous solution was prepared by delivering NO gas (99.99%; Tianjin Saiteer Special Gases Co., Tianjin, China) into deoxygenated and deionized water at 20 °C under an N_2_ atmosphere [32] and then diluting it with deoxygenated and deionized water to achieve the final concentration. The concentration of the NO solution was quantified using an Apollo 1000 free radical analyzer equipped with an ISO-NOPF200 flexible Nitric Oxide sensor (ISO-NOPF200; World Precision Instruments, Sarasota, FL, USA). (3) A 5 μmol L^−1^ potassium 2-(4-carboxyphenyl)-4,4,5,5-tetramethylimidazoline-1-oxyl-3-oxide (c-PTIO; Sigma, USA) solution, as an NO scavenger. (4) A 200 μmol L^−1^ l-NAME (N-nitro-l-arginine methyl ester) solution, as an inhibitor of NOS-like activity. (5) A 200 μmol L^−1^ sodium tungstate solution, as an inhibitor of nitrate reductase (NR). The concentrations of NO, c-PTIO, l-NAME, and sodium tungstate were chosen based on the results of previous reports [33,34]. Each treatment was repeated three times using 7 lots, with 30 peaches in each lot. Peaches were dried in air, and then stored at 0 °C at a relative humidity of 90%. The peaches were sampled every week, and 1 lot was randomly chosen from each repetition of each treatment. The peaches before treatments were sampled as time point 0 week.

### 2.2. Determination of the Rates of Respiration, Ethylene Production, Water Loss, the Content of Soluble Sugar, and Browning Degree

For the measurement of the respiration rate, 15 peaches from each treatment were randomly selected, and lots of 5 fruit were weighed and then sealed in a 10 L chamber with a rubber septum at 0 °C for 20 min. The three chambers were regarded as three replicates. The changes in CO_2_ content in the chamber were determined using an SY-1022 gas analyzer (Shiya Technology Co. Shijiazhuang, China). After that, the peaches were immersed in water in a bucket. The volume of water that was remained from the bucket was recorded. The respiratory rate was calculated by dividing the amount of CO_2_ by the difference between the volumes of the chamber and the peaches and expressed as μmol CO_2_ g^−1^ h^−1^ on the basis of fresh weight. 

For the measurement of ethylene production, a 5 mL sample of the headspace gas was withdrawn using a gas-tight syringe above each chamber through a septum stopper. The ethylene production was determined using a GC-9A gas chromatograph fitted with a GDX-502 column and an FID detector [35]. The column and injection temperatures were 70 and 120 °C, respectively. The rate of ethylene production was expressed as μmoL g^−1^ h^−1^ on the basis of fresh weight. 

For the measurement of the weight loss ratio, the weights of peaches were monitored weekly using an electronic analytical balance (BS124s, Sartorius, Germany). The water loss ratio was calculated according to the following formula: Weight loss ratio (%) = (original weight − weight after storage) × 100/original weight. 

For the measurement of the soluble sugar content (SSC), peach mesocarps (5 g) were homogenized in 15 mL distilled water and then boiled for 15 min. After cooling, the homogenate was centrifuged at 12,000× *g* and 4 °C for 20 min. The SSC of peaches was determined using the sulfuric acid anthrone method [36]. The supernatants (500 μL) were mixed well with 2 mL of distilled water, 0.5 mL of a 0.1 mol L^−1^ anthrone solution, and 5 mL of concentrated sulfuric acid, and then boiled for 10 min. The absorbance at 630 nm was recorded using a UV-2450 spectrophotometer (Shimadzu, Japan). The SSC was calculated by the standard curve of glucose and expressed as mg g^−1^ on the basis of fresh weight. 

For the measurement of the browning degree (BD), peach mesocarps (5 g) were homogenized in 15 mL of precooled 95% (*v/v*) ethanol. After incubation at 4 °C for 6 h, the homogenate was centrifuged at 12,000× *g* and 4 °C for 20 min. The absorbance at 410 nm was assayed using a UV-2450 spectrophotometer (Shimadzu, Kyoto, Japan) [37]. The BD was expressed as the absorbance at 410 nm (OD 410 nm).

### 2.3. Determination of the Contents of Endogenous H_2_S, Total Sulfhydryl, Cys, and Sulfite

For the measurement of H_2_S content, peach mesocarps (5 g) were homogenized in 10 mL of a precooled 50 mmol L^−1^ phosphate buffer (pH 6.8, containing 100  mmol L^−1^ EDTA and 200  mmol L^−1^ ascorbic acid), and the homogenate (5 mL) was transferred into a test tube, which was sealed with a rubber serum bottle cap and Parafilm and contained 5 mL of 1 mmol L^−1^ zinc acetate. Then, 5 mL of 1 mol L^−1^ HCl was injected with a syringe into the test tube through the rubber serum bottle cap. After incubation at 25 °C for 30 min, 1.5 mL of 5 mmol L^−1^ dimethyl-*p*-phenylenediamine (in 3.5 mol L^−1^ H_2_SO_4_) and 1.5 mL of 50 mmol L^−1^ ferric ammonium sulfate (in 100 mmol L^−1^ H_2_SO_4_) were injected into the tube in turn. After incubation at 25 °C for 15 min, the content of endogenous H_2_S in peaches was determined by the formation of methylene blue from dimethyl-*p*-phenylenediamine [38,39]. The absorbance of the reaction solution at 667 nm was recorded using a UV-2450 spectrophotometer (Shimadzu, Japan). The same procedures, without the zinc acetate solution, were used as the blanks. The content of H_2_S was calculated by a calibration curve of L^−1^ known concentrations of Na_2_S and expressed as μmol per gram of protein. The protein content was determined using Coomassie Brilliant Blue G-250, with bovine serum albumin as the standard [40]. 

For the measurement of the Cys content, peach mesocarps (0.5 g) were homogenized with 1 mL of an extraction buffer. The homogenates were centrifuged at 12,000× *g* and 4 °C for 10 min. The supernatants (40 μL) were mixed with 160 μL assay buffer of the cysteine assay kit (Solarbio, China), according to the manufacturer’s instructions, then incubated at room temperature for 15 min. The absorbance at 600 nm was recorded using a UV-2450 spectrophotometer (Shimadzu, Japan). The content of Cys in peaches was calculated from a standard curve of the known concentrations of Cys and expressed as mmol per gram of protein.

For the measurement of sulfite content, peach mesocarps (10 g) were homogenized with 15 mL distilled water and then mixed with 8 mL of a sodium tetrachloromercurate solution, which was produced by mixing 13.6% (*w/v*) HgCl_2_ and 6% (*w/v*) NaCl. After incubation at 4 °C for 8 h, the mixture was added to 2 mL of 250 mmol L^−1^ K_4_Fe(CN)_6_·3H_2_O and 2 mL of 1 mol L^−1^ zinc acetate dehydrate to clarify the solution. After centrifugation at 12,000× *g* and 4 °C for 20 min, the supernatants were collected. The supernatants (5 mL) were mixed with 1 mL of 100 mmol L^−1^ ammonium sulfamate, 1 mL of 100 mmol L^−1^ formaldehyde, and 1 mL of 2 g L^−1^ *p*-rosaniline hydrochloride and then incubated at room temperature for 20 min. The absorbance at 550 nm was recorded using a UV-2450 spectrophotometer (Shimadzu, Japan). The content of sulfite was calculated using sulfur dioxide as the standard solution and expressed as mmol per gram of protein. 

For the measurement of the total sulfhydryl content, peach mesocarps (5 g) were homogenized with 15 mL of a 0.1 mol L^−1^ potassium phosphate buffer (pH 7, containing 10 mmol L^−1^ ethylenediaminetetraacetic acid). After centrifugation at 15,000× *g* and 4 °C for 20 min, the supernatant was collected. The assay mixture contained 100 μL of the supernatant, 1880 μL of extraction buffer (15 mL of 0.1 mol L^−1^ potassium phosphate buffer (pH 7), containing 10 mmol L^−1^ ethylenediaminetetraacetic acid) and 20 μL of 20 mmol L^−1^ 5,5′-dithiobis (2-nitrobenzoic acid), and it was incubated at room temperature for 2 min. The absorbance at 412 nm was recorded using UV-2450 spectrophotometry (Shimadzu, Japan) [41]. The content of total sulfhydryl was calculated using a standard curve of the known concentrations of NaHS and expressed as mmol per gram of protein.

### 2.4. Determination of the Contents of Endogenous l-Arginine, NO, and NO_2_^−^

For the measurement of l-arginine content, peach mesocarps (1 g) were homogenized in 2 mL of precooled 10% (*w/v*) trichloroacetic acid. The homogenate was centrifuged at 10,000× *g* and 4 °C for 10 min. The supernatant (350 μL) was mixed with 200 μL of 10% (*w/v*) NaOH, 150 μL of 0.15% (*w/v*) 1-naphthol, and 500 μL of 1% (*w/v*) sodium hypochlorite. The absorbance at 530 nm was recorded using a UV-2450 spectrophotometer (Shimadzu, Japan) [42]. The content of l-arginine was calculated using a standard curve of the known concentrations of l-arginine and expressed as μmol per gram of protein.

For the measurement of NO content, peach mesocarps (5 g) were homogenized in 5 mL of 50 mmol L^−1^ acetic acid (pH 3.6) containing 4% (*w/v*) zinc acetate. After centrifugation at 10,000× *g* and 4 °C for 10 min, the supernatant (500 μL) was mixed with 400 μL of the reagent from the Nitric Oxide Detection Kit (Nanjing Jiancheng Bioengineering Institute, Nanjing, China). This kit measures NO content based on nitrate reductase catalysis. Distilled water was used as the blank, and the 100 μmol L^−1^ nitrate solution from the kit was used as the standard. After incubation at 37 °C for 60 min, the mixture was added to 200 μL of reagent III and 100 μL of reagent IV. After vortex oscillation for 30 s, the mixture was incubated at room temperature for 40 min and then centrifuged at 4000× *g* and 4 °C for 10 min. The supernatant (800 μL) was mixed well with 600 μL of the Color Reagent and incubated at room temperature for 10 min. The optical density (OD) at 550 nm was recorded using a UV-2450 spectrophotometer (Shimadzu, Japan). The content of NO was calculated by the following equation and expressed as μmol per gram of protein. NO content (μmol per gram of protein) = (OD of the sample − OD of the blank)/(OD of the standard − OD of the blank) × concentration of the standard × dilution ratio/protein content. 

For the measurement of NO_2_*^−^* content, peach mesocarps (5 g) were homogenized with 1 mL of saturated borax and 5 mL of double-distilled water and then boiled for 15 min. After cooling, the homogenate was mixed with 2 mL of 0.25 mol L^−1^ potassium ferrocyanide and 2 mL of 1 mol L^−1^ zinc acetate. The mixture was centrifuged at 10,000× *g* and 25 °C for 10 min. The content of NO_2_*^−^* was determined using the acidic Griess reaction [43]. The supernatant (1.5 mL) was mixed with 1 mL of 1% (*w/v*) *p*-aminobenzenesulfonamide (in 1.5 mol L^−1^ HCl) and 1 mL of 0.2% (*w/v*) *N*-(1-naphthyl) ethylenediamine dihydrochloride. After incubation in the dark for 15 min, the absorbance at 540 nm was determined using a UV-2450 spectrophotometer (Shimadzu, Japan). The NO_2_^−^ content was calculated using a standard curve of the known concentrations of NaNO_2_ and expressed as μmol per gram of protein.

### 2.5. Determination of Nitrate Reductase (NR) and NOS-Like Activity

For the measurement of NR activity, peach mesocarps (5 g) were homogenized with 5 mL of 50 mmol L^−1^ HEPES–KOH (pH 7.5, containing 1 mmol L^−1^ dithiothreitol, 1 mmol L^−1^ EDTA, and 7 mmol L^−1^ cysteine). The homogenate was centrifuged at 4 °C and 10,000× *g* for 10 min. The supernatants (300 μL) were mixed with 50 mmol L^−1^ HEPES–KOH (pH 7.5, 100 μmol L^−1^ NADH, 5 mmol L^−1^ KNO_3_, and 6 mmol L^−1^ MgCl_2_) and incubated at 30 °C for 30 min. Then, 200 μL of 1% (*w/v*) sulfanilamide in 1.5 mol L^−1^ HCl and 200 μL of 0.2% (*w/v*) *n*-naphtylethylenediamine dihydrochloride, was added to terminate the reaction. After incubation for 15 min, the absorbance at 540 nm was recorded using a UV-2450 spectrophotometer (Shimadzu, Japan) [42]. The NR activity was expressed as nmol NO_2_^−^ per minute per gram of protein.

For the measurement of NOS-like activity, peach mesocarps (2 g) were homogenized with 1 mL of 50 mmol L^−1^ Tris–HCl (pH 7.4, containing 1 mmol L^−1^ EDTA, 320 mmol L^−1^ sucrose, 1 mmol L^−1^ dithiothreitol, 1 μmol L^−1^ pepstatin, and 1 mmol L^−1^ phenylmethanesulfonyl fluoride). The homogenate was centrifuged at 4 °C and 10,000× *g* for 10 min. The supernatants (400 μL) were mixed with 200 μL of 25 mmol L^−1^ Tris-HCl (pH 7.5, containing 1 μmol L^−1^ flavin mononucleotide, 1 mmol L^−1^ NADPH, 0.6 mmol L^−1^ CaCl_2_, and 2 mmol L^−1^ l-arginine), and incubated at 37 °C for 10 min. Then, 400 μL of deaerated 50 mmol L^−1^ HEPES (pH 5.5, containing 5 mmol L^−1^ EDTA) was added to terminate the reaction. The NOS-like activity was detected by the production of NO catalyzed by NOS-like, with l-arginine as the substrate [42], using a Nitric Oxide Detection Kit (Nanjing Jiancheng Bioengineering Institute, China). The NOS-like activity was expressed as nmol NO per minute per gram of protein.

### 2.6. Determination of the Activities of β-Cyanoalanine Synthase (β-CAS), Sulfite Reductase (SiR), l/d-Cysteine Desulfhydrase (L/D -CDes), and O-Acetylserine(thiol)lyase (OAS-TL)

For the measurement of β-CAS activity, peach mesocarps (10 g) were homogenized in 50 mL of an extraction buffer (0.1 mol L^−1^ Tris-HCl (pH 9.0), containing 1 mmol L^−1^ EDTA) in an ice bath. After filtering the homogenate through four layers of cheesecloth, the filtrate was centrifuged at 14,000× *g* and 4 °C for 15 min. The supernatant (1 mL) was mixed with 0.5 mL of 0.1 mol L^−1^ KCN (in an extraction buffer) and 0.5 mL of 50 mmol L^−1^ l-Cys (in an extraction buffer). After incubating at 30 °C for 60 min, the mixture was mixed with 1 mL of an acidic dye precursor reagent (15 mmol L^−1^ *N*,*N*-dimethyl-1,4-phenylenediamine dihydrochloride, 3 mmol L^−1^ ferric chloride, and 4.2 mol L^−1^ HCl). After standing at 30 °C for 30 min, the mixture was centrifuged at 14,000× *g* and 4 °C for 10 min. The supernatant was collected. The β-CAS activity was measured with the method of acid dimethyl *p*-phenylenediamine staining [44]. The absorbance at 745 nm was recorded using a UV-2450 spectrophotometer (Shimadzu, Japan). One unit of the enzyme activity was defined as the conversion of 1 μmol of cysteine into cyanoalanine and H_2_S per hour, under the stated assay conditions. The activity of β-CAS was calculated using the standard curve of sodium sulfide with known concentrations and expressed as μmol per minute per gram of protein.

For the measurement of the SiR activity, peach mesocarps (10 g) were homogenized in 10 mL of a 50 mmol L^−1^ HEPES–KOH buffer (pH 7.4), containing 10 mmol L^−1^ KCl, 1 mmol L^−1^ EDTA, 1 mmol L^−1^ EGTA, 10% (*v/v*) glycerin, 10 mmol L^−1^ DTT, and 0.5 mmol L^−1^ PMSF. The mixture was centrifuged at 14,000× *g* and 4 °C for 15 min. The supernatants were incubated at 55 °C for 90 s and then centrifuged at 14,000× *g* and 4 °C for 5 min. Then, they were collected. The reaction solution contained 1 mL of the supernatant and 5 mL of a 50 mmol L^−1^ HEPES–KOH buffer (pH 7.8), containing 1 mmol L^−1^ Na_2_SO_3_, 1 mmol L^−1^ EDTA, and 1 mmol L^−1^ NADPH, and was incubated at 30 °C for 10 min. Then, 100 μL of 30 mmol L^−1^ FeCl_3_ (in 1.2 mmol L^−1^ HCl) and 100 μL of 20 mmol L^−1^ *N*,*N*-Dimethyl-*p*-phenylenediamine dihydrochloride (DMPD) (in 7.2 mmol L^−1^ HCl) were added to terminate the reaction. After incubation for 15 min at room temperature, the absorbance at 670 nm was recorded using a UV-2450 spectrophotometer (Shimadzu, Japan) [2]. The SiR activity was calculated by a standard curve of Na_2_S and expressed as μmol per minute per gram of protein.

For the measurement of the L/D -CDs activity, peach mesocarps (5 g) were homogenized with 50 mL of 20 mmol L^−1^ Tris-HCl (pH 8.0). The homogenate was centrifuged at 12,000× *g* and 4 °C for 20 min. The reaction solution contained 1 mL of the supernatant and 1 mL of 100 mmol L^−1^ Tris-HCl (pH 9.0, containing 0.8 mmol L^−1^ l-cysteine and 2.5 mmol L^−1^ DTT). After incubation for 15 min at 37 °C, the reaction was terminated by adding 100 μL of 30 mmol L^−1^ FeCl_3_ (in 1.2 mol L^−1^ HCl) and 100 μL of 20 mmol L^−1^ DMPD (in 7.2 mol L^−1^ HCl). The activity of l/d-CDs was determined by the release of H_2_S from l-cysteine or d-cysteine in the presence of DTT [2,45]. The absorbance at 670 nm was recorded using a UV-2450 spectrophotometer (Shimadzu, Japan). The activity of l-CD was calculated by the standard curve of Na_2_S. The activity of d-CD was determined using the same procedure, with d-cysteine, instead of l-cysteine, and a Tris–HCl buffer (pH 8.0). The activities of l-/d-CDs were expressed as μmol per minute per gram of protein.

For the measurement of the OAS-TL activity, peach mesocarps (10 g) were homogenized with 30 mL of a 50 mmol L^−1^ phosphate buffer (pH 7.5, containing 10 μmol L^−1^ pyridoxal-phosphate and 1 mmol L^−1^ dithiothreitol). The homogenate was centrifuged at 12,000× *g* for 15 min at 4 °C. The reaction solution contained 1 mL of the supernatant and 1 mL of 100 mmol L^−1^ HEPES–KOH (pH 7.2, containing 10 mmol L^−1^ *O*-acetyl-l-serine, 5 mmol L^−1^ DTT, and 5 mmol L^−1^ Na_2_S) and was incubated at 50 °C for 10 min. The reaction was stopped by adding 1 mL of 20% (*v/v*) trichloroacetic acid. After centrifugation at 13,000× *g* for 20 min at 4 °C, the supernatant was mixed with 1 mL of glacial acetic acid and 2 mL of a ninhydrin solution (0.25 g of ninhydrin in 6 mL of glacial acetic acid and 4 mL of concentrated hydrochloric acid). After incubation at 100 °C for 10 min, the mixture was cooled in an ice bath and then mixed with 5 mL of ethanol. The absorbance at 560 nm was detected using a UV-2450 spectrophotometer (Shimadzu, Japan) [46,47]. The activity of OAS-TL was expressed in units that correspond to the formation of 1 µmol of cysteine per minute per gram of protein, with a calibration curve for the known concentrations of l-cysteine. 

### 2.7. Statistical Analysis

The experiments were performed in a completely randomized fashion, and each experiment was repeated three times. The data are reported as the mean ± standard error (SE) and were processed by an analysis of variance (ANOVA), with *p* < 0.05 indicating significant differences, based on the least significant difference (LSD) test.

## 3. Results

### 3.1. Changes in the Rates of Respiration, ethylene Production, Weight Loss, Soluble Sugar Content, and Browning Degree

**Figure 1:**
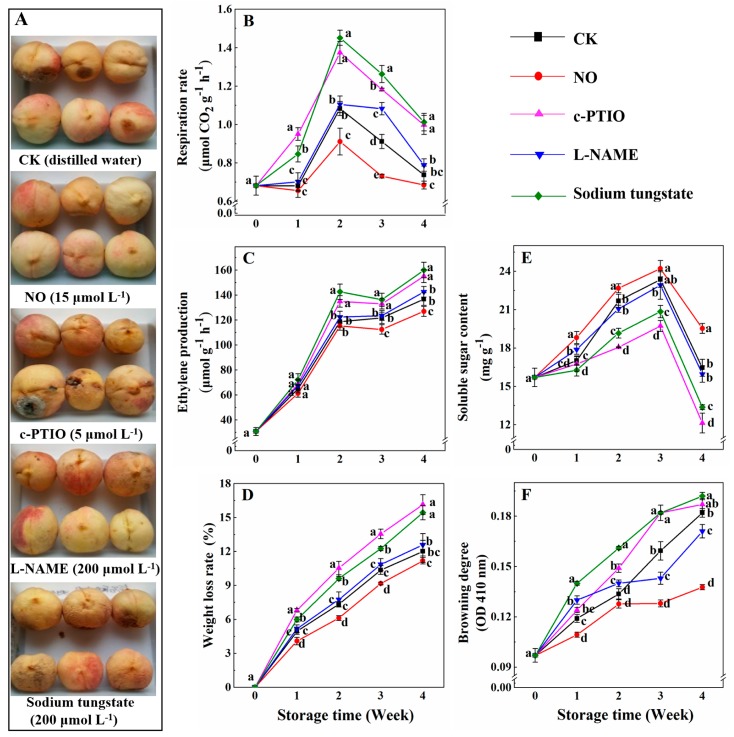
Appearance of peaches after different treatments at week 4 of cold storage (**A**); Changes in the rates of respiration (**B**) and ethylene production (**C**), weight loss (**D**), soluble sugar content (**E**), and browning degree (**F**) of peaches, after treatments and during cold storage. Each experiment was repeated three times, and the rate was expressed as the mean ± standard error (SE). Different letters for each week indicate a significant difference (*p* = 0.05) between the treatments.

Figure 1A shows the peach fruits after different treatments at week 4 of storage. Compared with other treatments, exogenous NO maintained the peach fruits during storage, and they exhibited good external attributes. Compared with the control, exogenous NO significantly reduced the respiration rate, ethylene production, weight loss rate, and browning degree, and it allowed the peaches to maintain a high content of soluble sugar during storage (Figure 1). Especially, at week 3, the respiration rate, ethylene production, weight loss rate, and browning degree of peaches treated with NO were still 80.21%, 92.40%, 88.51%, and 80.34%, respectively, compared with the control. No differences were found in the ethylene production, weight loss, and content of soluble sugar between peaches treated with l-NAME and the control peaches. The respiration rate of peaches treated with l-NAME was 1.22 times that of the control at week 3, and no significant difference was found for other weeks. Both c-PTIO and sodium tungstate promoted the rates of respiration, ethylene production, and weight loss, and decreased the soluble sugar content in peaches during storage. At week 3, the respiration rate, ethylene production, and weight loss rate of peaches treated with c-PTIO or sodium tungstate were respectively about 1.30 and 1.39, 1.10 and 1.12, and 1.31 and 1.18 times higher than the those of the control, while the soluble sugar content was only 84.45% and 89.29% of the control. The browning degree of peaches treated with l-NAME was higher before week 2, and then lower than that of the control. However, the browning degree of peaches treated with c-PTIO remained higher than that of the control during storage (Figure 1F).

### 3.2. Changes in the Contents of Endogenous H_2_S, Cys, Sulfite, and Total Sulfhydryl

**Figure 2:**
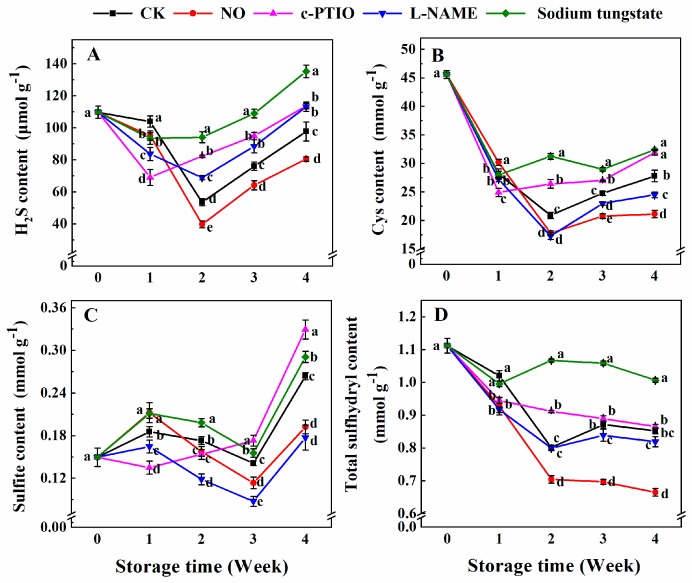
Changes in the contents of endogenous H_2_S (**A**), Cys (**B**), sulfite (**C**), and total sulfhydryl (**D**) in peaches after treatment and during cold storage. Each experiment was repeated three times, and the rate was expressed as the mean ± standard error (SE). Different letters for each week indicate a significant difference (*p* = 0.05) between the treatments.

The H_2_S content of peaches treated with NO was significantly lower than that of the control during storage (Figure 2A). Especially at week 3, the H_2_S content in peaches treated with NO was only 66.3% that of the control. Compared with the control, the treatments with c-PTIO, l-NAME, and sodium tungstate decreased the H_2_S content in week 1 and then significantly increased the H_2_S content in peaches after week 2. The H_2_S content in peaches treated with sodium tungstate was significantly higher than that of the peaches subjected to the other treatments after week 2.

The content of Cys in the control peaches rapidly decreased in the first 2 weeks and then increased slowly (Figure 2B). The Cys content in peaches treated with NO was 1.11 times that of the control in week 1 and only 85.14%, 83.87%, 76.02% of the control in weeks 2, 3 and 4, respectively. The Cys content in peaches treated with c-PTIO was lower at week 1 and then significantly higher than that of the control. The Cys content in peaches treated with l-NAME was lower than that of the control during storage. The Cys content in peaches treated with sodium tungstate was higher than that of the control after week 2, and was higher at weeks 2 and 3 than that of peaches treated with c-PTIO.

The contents of sulfite in peaches treated with exogenous NO were higher, at week 1, and then lower than that of the control (Figure 2C). The sulfite content in peaches treated with c-PTIO was significantly lower, at weeks 1 and 2, and higher at weeks 3 and 4 than that of the control. The sulfite content in peaches treated with l-NAME was lower than that of the control during storage. Peaches treated with sodium tungstate maintained a higher sulfite content than the control during storage.

The total sulfhydryl content in peaches treated with NO remained lower than that of the control during storage (Figure 2D). The total sulfhydryl content in peaches treated with c-PTIO was 92.61% of the control, at week 1, and 1.14 times of the control at week 2. At week 1, the total sulfhydryl content in peaches treated with l-NAME was 89.88% of the control. Sodium tungstate allowed peaches to maintain a significantly higher content of total sulfhydryl than the control and peaches subjected to the other treatments after week 1. The total sulfhydryl content of peaches treated with sodium tungstate was 1.33, 1.27, and 1.24 times that of the control at weeks 2, 3, and 4, respectively.

### 3.3. Changes in the Contents of Endogenous l-Arginine, NO, and NO_2_^−^

**Figure 3:**
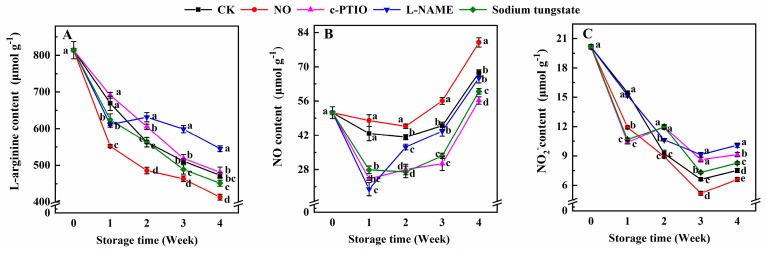
Changes in the contents of l-arginine (**A**), endogenous NO (**B**), and NO_2_^–^ (**C**) in peaches after treatment and during cold storage. Each experiment was repeated three times, and the rate was expressed as the mean ± standard error (SE). Different letters at each week indicate a significant difference (*p* = 0.05) between the treatments.

The endogenous l-arginine content of peaches treated with NO was lower than that of the control during storage (Figure 3A). The l-arginine content of peaches treated with c-PTIO was 1.08 and 1.07 times that of the control at weeks 2 and 3, respectively. The l-arginine content of peaches treated with l-NAME was 91.34% of the control at week 1, and 1.12, 1.47, and 1.13 times that of the control at weeks 2, 3, and 4, respectively. The l-arginine content in peaches treated with sodium tungstate was also lower than that of the control during storage, except at week 2. The l-arginine content of peaches treated with sodium tungstate at weeks 1 and 4 was 93.32% and 95.46% of the control, respectively. 

Compared with the control, peaches treated with exogenous NO maintained a higher content of NO during storage (Figure 3B). The NO content in peaches treated with c-PTIO or sodium tungstate was significantly lower than that of the control during storage. The NO content in peaches treated with l-NAME was 46.77% and 90.12% of the control at weeks 1 and 2, respectively. No significant difference in the NO content between the control peaches and peaches treated with l-NAME was found after week 3.

The NO_2_^−^ content of the control peaches decreased before week 3, with a transient increase at week 4 (Figure 3C). The NO_2_^−^ content of NO-treated peaches was 77.16%, 78.11%, and 87.79% of the control at weeks 1, 3, and 4, respectively. Compared with the control, c-PTIO and sodium tungstate allowed peaches to maintain a lower NO_2_^−^ content at week 1, and a higher NO_2_^−^ content after week 2. The NO_2_^−^ content of peaches treated with l-NAME was also higher than that of the control in week 2.

### 3.4. Changes in the NR and NOS-Like Activities

**Figure 4:**
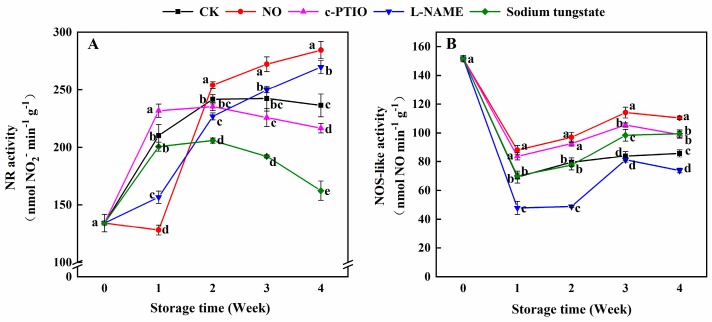
Changes in the nitrate reductase (NR) (**A**) and nitric oxide synthase (NOS)-like activities (**B**) in peaches after treatment and during cold storage. Each experiment was repeated three times, and the rate was expressed as the mean ± standard error (SE). Different letters in each week indicate a significant difference (*p* = 0.05) between the treatments.

Compared to the control, the NR activity of peaches treated with exogenous NO was lower at week 1 and higher after week 2 (Figure 4A). The NR activity of peaches treated with NO was only 63.85% at week 1, but 1.20 times that of the control at week 4. Compared with the control, peaches treated with c-PTIO maintained a higher NR activity at week 1 and a lower NR activity after week 3. l-NAME exhibited the opposite effect on the NR activity against c-PTIO. Sodium tungstate significantly inhibited the NR activity in peaches after week 2. The NR activity of peaches treated with sodium tungstate was 85.21%, 76.34%, and 68.65% of the control in weeks 2, 3, and 4, respectively.

The NOS-like activity of peaches treated with NO was significantly higher than that of the control during storage (Figure 4B). c-PTIO also allowed peaches to maintain a higher NOS-like activity than that of the control during storage. The NOS-like activity of peaches treated with l-NAME was significantly lower than that of the control during storage, except at week 3. Before week 2, there was no significant difference observed in the NOS-like activity between the control peaches and peaches treated with sodium tungstate, while the NOS-like activity of peaches treated with sodium tungstate was 1.17 and 1.16 times that of the control at weeks 3 and 4, respectively. 

### 3.5. Changes in the Activities of l-CD, d-CD, β-CAS, OAS-TL, and SiR

**Figure 5:**
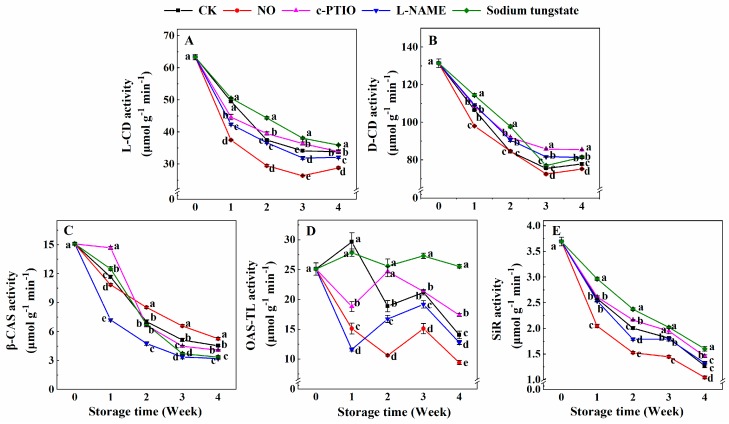
Changes in the activities of l-CD (**A**), d-CD (**B**), β-CAS (**C**), OAS-TL (**D**), and SiR (**E**) in peaches, after treatment and during cold storage. Each experiment was repeated three times, and the rate was expressed as the mean ± standard error (SE). Different letters in each week indicate a significant difference (*p* = 0.05) between the treatments.

The l-CD activity of peaches treated with NO was significantly lower than that of the control during storage (Figure 5A). The l-CD activity of peaches treated with c-PTIO was 90.02% of the control at week 1, and was higher than that of the control at weeks 2 and 3. The l-CD activity of peaches treated with l-NAME was lower than that of the control but higher than that of peaches treated with NO. Sodium tungstate allowed peaches to maintain a higher l-CD activity than that of the control after week 2.

The d-CD activity of peaches treated with NO was 92.00% and 94.54% of the control in weeks 1 and 4, respectively (Figure 5B). Both c-PTIO and l-NAME allowed peaches to maintain a higher d-CD activity than that of the control after week 2. The d-CD activity of peaches treated with sodium tungstate was also higher than that of the control before week 2.

The β-CAS activity of peaches decreased gradually during storage (Figure 5C). The β-CAS activity of peaches treated with NO was 93.05% of the control at week 1, and then significantly higher than that of the control after week 2. The β-CAS activity of peaches treated with c-PTIO was 1.26 times that of the control at week 1, and 87.45% and 89.88% that of the control in weeks 3 and 4, respectively. Sodium tungstate exhibited an effect on the β-CAS activity that was similar to that of c-PTIO. Compared with the control, l-NAME allowed peaches to maintain a lower β-CAS activity during storage. 

Both NO and l-NAME allowed peaches to maintain lower OAS-TL activity than that of the control during storage (Figure 5D). The OAS-TL activity of NO-treated peaches was 50.96%, 56.29%, 71.21%, and 67.04% of the control at weeks 1, 2, 3, and 4, respectively. At the same time, the OAS-TL activity of peaches treated with l-NAME was 39.17%, 88.68%, 90.38%, and 90.98% of the control at weeks 1, 2, 3, and 4, respectively. Sodium tungstate allowed peaches to maintain a higher OAS-TL activity than that of the control after week 2. The OAS-TL activity of peaches treated with sodium tungstate was 1.36, 1.29, and 1.82 times that of the control at weeks 2, 3 and 4, respectively. The OAS-TL activity of peaches treated with c-PTIO was 63.39% of the control at week 1, and 1.31 and 1.24 times that of the control at weeks 2 and 4, respectively.

The SiR activity of the control peaches decreased during storage (Figure 5E). The SiR activity of peaches treated with NO was significantly lower than that of the control during storage. Moreover, the SiR activity of peaches treated with sodium tungstate was significantly higher than that of the control during storage. The SiR activity of peaches treated with c-PTIO was also higher than that of the control after week 2. There was no significant difference in the SiR activity of peaches between the control peaches and peaches treated with l-NAME during storage, except in week 2. In week 2, the SiR activity of peaches treated with l-NAME was 89.27% of the control.

## 4. Discussion

Both NO and H_2_S are involved in regulating the ripening of fruits [26,48]. Exogenous NO decreased the contents of H_2_S, Cys, and total sulfhydryl and the activities of l-CD, d-CD, β-CAS, OAS-TL, and SiR in peaches during storage. Partially opposite results were found for peaches treated with c-PTIO, l-NAME, and sodium tungstate. These results suggest that exogenous NO could retard endogenous H_2_S metabolism in peaches during cold storage. However, pretreatment with the NO donor sodium nitroprusside (SNP) enhances the activity of l-CD, which, in turn, induces accumulation of endogenous H_2_S in maize seedlings [49]. NO induces H_2_S production by regulating the key enzymes involved in biosynthesis and degradation of H_2_S in soybean [50]. Nitrate reductase-dependent NO production is involved in H_2_S-induced nitrate stress tolerance in tomato seedling [51]. The conflict might depend on the difference between the plants and the fruits. Endogenous H_2_S is up-regulated during sweet pepper fruit ripening [52]. The homeostasis of endogenous H_2_S is responsible for the alleviation of senescence of postharvest daylily flower [22]. Co-treatment of NaHS and SNP indicates the synergistic function of H_2_S and NO effectively prolongs the shelf life and reduces the decay rate of a harvested strawberry [53]. These support the idea of a possible interplay between H_2_S and NO in postharvest life extension [9]. However, the relationship between NO and H_2_S in peaches, as a climacteric fruit, is still not clear during storage.

**Figure 6:**
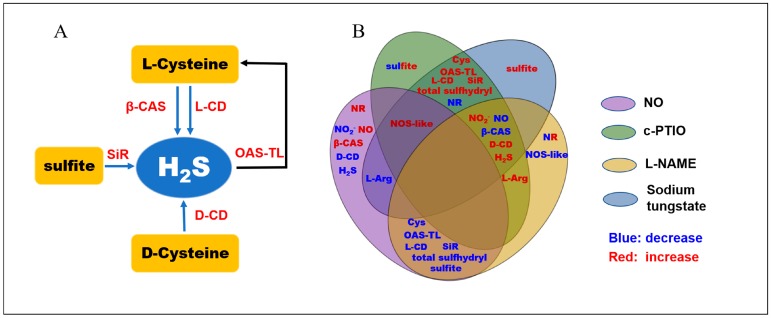
(**A**) H_2_S metabolism pathways in plants. (**B**) Venn diagram showing the interrelationship between the biosynthesis of NO and the metabolism of H_2_S in peach fruit under different treatments. The sets with various fill colors present different treatments. The blue or the red fonts within sets respectively indicate a decrease or increase of the parameters under the corresponding treatments.

To reveal the possible interrelationship between the biosynthesis of NO and the metabolism of H_2_S in peach fruit under different treatments, information concerning the changes of parameters is visually represented in Figure 6. l-CD and d-CD catalyze the degradation of l-cysteine and d-cysteine, respectively, to produce H_2_S [2]. Exogenous NO decreased the activities of l-/d-CDs, which reduced the H_2_S production due to cysteine. In plants, SiR, which functions in the sulfate assimilation pathway, can reduce sulfite to produce H_2_S, with ferredoxin as an electron donor. It is involved in cold and oxidative stress tolerance, possibly due to its modulation of sulfite reduction and GSH-dependent H_2_O_2_ scavenging [54]. Exogenous NO significantly promoted the decrease of SiR activity in peaches, which could contribute to the low content of H_2_S in peaches treated with exogenous NO during cold storage. β-CAS is an enzyme that catalyzes the conversion of cysteine and cyanide to β-cyanoalanine and H_2_S [55]. However, exogenous NO increased the activity of β-CAS in peaches during storage, which conflicted with the decrease in H_2_S content. β-CAS would also not be a major contributor to the low content of H_2_S in peaches during cold storage. OAS-TL, also known as *O*-acetylserine sulfhydrylase and cysteine synthase, catalyzes the synthesis of cysteine from sulfide and *O*-acetylserine (OAS) and reduces the H_2_S content, and the reverse reaction releases H_2_S [2]. Compared with the control and other treatments, exogenous NO inhibited OAS-TL activity, which led to the low content of Cys in peaches during storage. Exogenous NO decreased H_2_S content mainly by reducing the activities of l-/d-CDs, OAS-TL, and SiR.

The effects of c-PTIO, as a NO scavenger, on the activities of enzymes in the H_2_S metabolism were partially opposed to those of exogenous NO. These results also confirmed the effects of NO on H_2_S metabolism in peaches. In the leaves of *Arabidopsis*, c-PTIO prevents H_2_S production and l-/d-CD activity [56], which is in accordance with the present results at week 1 but opposite to those after week 2. This opposing result might be due to the differences between leaves of *Arabidopsis* and peach fruits during storage. 

l-NAME, as an inhibitor of NOS-like activity, decreased NO content in peaches. In tomatoes, NO accumulation caused by NaHS is altered shortly after l-NAME is added [51]. l-NAME also decreases the DII-VENUS fluorescence caused by H_2_S toxicity in the roots of *Arabidopsis* [57]. l-NAME has no significant effect on ethylene-induced H_2_S biosynthesis in *Vicia faba* L. [58]. The different experimental materials led to the differences in the roles of l-NAME on H_2_S. Inhibiting NOS-like activity improved the H_2_S content of peaches, possibly by increasing the d-CD activity, but not the l-CD activity, and by inhibiting the activity of OAS-TL in the process of H_2_S degradation.

As an inhibitor of NR, sodium tungstate significantly increased the contents of H_2_S, Cys, sulfite, and total sulfhydryl and delayed the decrease in the activities of l-/d-CDs, OAS-TL, and SiR. Inhibiting NR activity improved H_2_S metabolism in peaches after week 2 of storage by promoting the degradation of cysteine and the reduction of sulfite. As the major source of endogenous NO in plants, NR or the NR pathway of NO biosynthesis should be involved in regulating H_2_S metabolism in peaches during cold storage. It has been reported that NR-dependent NO production is involved in H_2_S-induced nitrate stress tolerance in tomato [51]. H_2_S generated by l-CD also mediates nitrate reductase-generated NO in *Arabidopsis* [59]. This evidence indicates an interplay between H_2_S metabolism and NR-dependent NO biosynthesis in peaches during cold storage. There should be some overlap between the functions of NO and H_2_S under both physiological and adverse conditions [10]. 

In conclusion, exogenous NO, c-PTIO, l-NAME, and sodium tungstate regulated H_2_S metabolism in peaches during storage through different mechanisms. While promoting the activity of β-CAS, exogenous NO decreased the content of H_2_S, Cys, sulfite, and total sulfhydryl, mainly by inhibiting the catalysis of SiR, l/d-CDs, and OAS-TL. SiR, l/d-CDs, and OAS-TL, rather than β-CAS, contributed to the increase of the H_2_S content of peaches treated with c-PTIO. l-NAME increased the H_2_S content mainly by allowing peaches to maintain a high activity of d-CD. The effect of sodium tungstate on the H_2_S metabolism of peaches was similar to that of c-PTIO. 

In this study, exogenous NO maintained high NO content and inhibited H_2_S content in peaches during storage, but c-PTIO-, l-NAME-, and sodium tungstate exhibited the opposite effects on the H_2_S metabolism against exogenous NO. These results imply that there is crosstalk between NO and H_2_S, and the regulation of the H_2_S metabolism by NO scavengers and NO synthesis inhibitors might depend on different mechanisms. However, further research needs to be conducted to clarify the relationship between, and the effects of, NO and H_2_S in the ripening of fruits. 

## 5. Conclusions

Exogenous NO decreased endogenous H_2_S metabolism, while c-PTIO, l-NAME, and sodium promoted H_2_S metabolism in peaches during cold storage. The regulation on H_2_S metabolism in peaches by exogenous NO, NO scavenger, and the inhibitors of NO synthesis were different.
